# Brood size in an uncertain world

**DOI:** 10.1098/rsos.221362

**Published:** 2023-06-21

**Authors:** Alex James, Alexander Hann, E. Penelope Holland

**Affiliations:** ^1^ School of Maths and Stats, University of Canterbury, Christchurch, New Zealand; ^2^ Department of Biology, University of York, York, UK

**Keywords:** masting, reproduction, stochastic model, optimal brood size

## Abstract

Reproduction in an uncertain world is fraught. The consequences of investing in too many offspring in a resource poor season can be disastrous but so too is missing the opportunity of a resource rich year. We consider a simple population and individual growth model and use Lyapunov exponents to find analytical results for the optimum brood size under stochastic environmental conditions. We show that if the environment shows dramatic changes between breeding seasons choosing a smaller brood size is more likely to be successful but the best strategy is to synchronize your reproduction to the food availability. Finally, we show that if the cost of having offspring is high it can be better to live in a highly varying world with a plastic strategy that synchronizes to the environment than to live in a deterministic world with a constant strategy, a finding with implications for invasive species and climate change.

## 1. Introduction

The evolution of an optimal brood size has been an area of study for many years [1,2], in mammals (litter size), birds (clutch size) and plants (seed production). The existence of an optimal reproductive output is intuitively simple. If an organism always produces few offspring, each one must have a good chance of survival (for example, with parental care from a K-strategist animal) in order to result in population stability or growth. Conversely, if organisms produce many offspring, there is likely to be low parental investment in offspring (in animals) and high competition so the survival probability of each individual is low but there is a high probability of some surviving. An intermediate number balances these two options and gives an optimal brood or litter size [3].

The exact size of this optimal brood depends on many factors which affect the survival and fecundity of individuals at different ages and classes including latitude, day length, temperature, resource limitation and predation [4], and of course, how ‘optimal’ is defined in relation to the population. There are a number of models that explore these ideas and consider conflict with respect to the optimal offspring size from the viewpoint of both the parent and the offspring [4–7]. From the parents' perspective, having offspring requires a trade-off of investment in reproduction and survival [8–10]. If reproduction, e.g. seed production [11], pregnancy and lactation [9] or parental care [12], is related to increased parent mortality, it may be optimal to have fewer offspring now, in order to increase the chance of having further broods in the future. Offspring from smaller broods may also be more viable, e.g. as a result of larger body or seed sizes. Parental allocation of investment to offspring also differs as the quality of the current mate varies [13] and this differential allocation of offspring resources can be highly partner dependent [14]. From the offsprings’ perspective, a higher individual survival rate is key. General results include that higher per capita juvenile mortality and scarcer food lead to smaller brood sizes (defined as the number of offspring at birth in the animal kingdom, or viable seeds released in the plant kingdom) [15].

Animals and plants often live in environments where resources to facilitate reproduction fluctuate across years, and their populations may fluctuate in response to these. For example, temperature and precipitation in the previous 1–3 years are strong drivers of variable, synchronous plant reproduction e.g. Kelly *et al*. [16], resulting in mast seed events that in turn drive irruptive population dynamics in seed consumers [17] and their predators [18]. When the environment is favourable, it might be advantageous to have more offspring per brood, and/or multiple broods to make the most of ephemeral resources. By contrast, smaller reproductive output (smaller brood sizes) could be expected in years when resources are less available. Many populations follow this pattern; for example, mast seeding driven by environmental cues [16]. A well-known example from the animal kingdom is the highly endangered New Zealand kākāpō (*Strigops habroptilus*), which lay a single egg at the start of mast years. This long-lived bird can anticipate a mast year and prepare to mate months before any obvious signals [19]. Another example is the red squirrel (*Sciurus vulgaris*) who may also anticipate mast years, producing larger litter sizes in the spring of those years [3].

Bonsall & Klug [20] looked at the evolution of parental care in stochastic environments and concluded that increasing parental care can be beneficial in a varying environment. There is also a wide body of research on plastic breeding in response to fluctuating environments that primarily focuses on the genetic aspects often using the theory of evolutionary stable strategies, for example [21–25]. However, there are few studies that directly consider the effects of environmental stochasticity on brood size, e.g. the number of babies born or hatched, or seeds leaving the plant. We present a model to explore the trade-offs between parental investment in reproduction, and juvenile growth, with respect to optimal brood size. We examine strategies that could evolve to optimize population growth under different environmental conditions, and ask how these differ when individuals are able to take advantage of annual changes in resource availability and consequent juvenile growth rates. We explore model parameter space to assess situations in which it is advantageous to live in a changing environment rather than a constant one, and use real-world scenarios to reflect on the consequences for species experiencing invasions or climate change.

## 2. Model overview

We present the model in two parts. First, we develop the deterministic juvenile growth and mortality model based on von Bertalanffy growth and size-dependent mortality. This is coupled with the discrete population map that includes adult mortality. We define for the ‘best’ strategies as follows: for the offspring, the best strategy is that in which juveniles from a single brood have the highest chance of survival; for the population (parent and offspring combined), the best strategy is that in which the population grows fastest.

We explore the effect of stochasticity on brood size in two ways. First we include stochasticity via individual variation in brood size across the parent population, and then add environmental variation by changing resource availability across years. We search for the best strategies in a deterministic, static environment and in a stochastic environment.

## 3. Offspring strategy

We start by assuming a simple model of individual juvenile growth which uses differential equations and follows the von Bertalanffy growth curve. Juvenile mortality proportional to the number of currently surviving offspring and offspring size is then included. We make the deliberately simple assumption that juvenile growth rate is inversely related to the initial number of offspring. The key model output is the number of surviving offspring at the end of the juvenile growth period.

A mature individual has *B*_0_ offspring. The size of a juvenile, *L*(*t*), where time *t* is likely measured in days follows a von Bertalanffy growth curve starting at size *L*_0_ and reaching maturity at a threshold size *L*_∞_ before the next breeding season, i.e. generations do not overlap. Juveniles have growth rate *r*

3.1 $$\frac{dL(t)}{dt}=r(L_{\infty }-L(t))$$

with size

3.2 $$L(t)=L_{\infty }(1-\exp(-rt))+L_{0}\exp(-rt).\;$$

Without loss of generality we measure *L*(*t*) as a fraction of the final size i.e. *L*_∞_ = 1. Juveniles are subject to an external mortality *μ*_J_ which reduces with size but is non-zero for adults. The number of juveniles alive at time *B*(*t*) thus follows the differential equation

3.3 $$\frac{dB}{dt}=-\mu_{J}(1-L(t))B\;\;\mathrm{with}\;B(0)=B_{0}.\;$$

By substituting the solution for *L*(*t*) (equation (2.2)) into the differential equation for *B*(*t*) (equation (2.3)) we can solve using separation of variables to give

3.4 $$B(t)=B_{0}\exp\left(\frac{-\mu_{j}(1-L_{0})(1-\exp(-rt))}{r}\right)\;.$$

Hence, the number of offspring that survive to reach maturity is

3.5 $$B_{\infty }=\underset{t\to \infty }{\lim}B(t)=B_{0}\exp\left(-\frac{\mu_{J}(1-L_{0})}{r}\right).$$

We assume that, by having fewer offspring, the parent is able to confer an advantage on the offspring through an increased juvenile growth rate i.e. $r=r_{0}/B_{0}$. This represents parental care (in animals) and/or greater investment in the viability/quality of the individual offspring by animals and plants. The number of survivors is then

3.6 $$B_{\infty }=B_{0}\exp\left(-\frac{\mu_{J}B_{0}(1-L_{0})}{r_{0}}\right).\;$$

To illustrate, for the example parameters given in table 1, the expected number of survivors for a brood size of *B*_0_ = 1 is *B*_∞_ = 0.67. This increases to 0.91 for a brood size of 3 (close to the optimum) and decreases to only 0.19 survivors for very large broods of 10.

**Table 1.** Example parameter values used to illustrate the results. We assume time is measured in days and breeding seasons are annual but the results generalize without specific units.

description	parameter	value
juvenile growth rate	*r*	0.5 (time^−1^)
juvenile mortality	*μ*_J_	0.2 (time^−1^)
birth size	*L*_0_	0.01
intrinsic adult mortality	*μ*_A0_	0.07 (year^−1^)
adult mortality due to brood size costs	*α*	varied
food availability coefficient of variation	CV	varied
population growth rate	*R*_0_	model output

The brood size $B_{0}^{*}$ which maximizes the number of survivors $B_{\infty }^{*}$ is

3.7 $$B_{\infty }^{*}=\frac{r}{\mu_{J}(1-L_{0})}\exp\left(\frac{1-L_{0}}{L_{0}-1}\right)\;\;\;\mathrm{at}\;B_{0}^{*}=\frac{r_{0}}{\mu_{J}(1-L_{0})}.\;$$

This is by definition the optimal brood size strategy for the offspring, i.e. $B_{0}^{*}$ maximizes the number of offspring surviving to $B_{\infty }^{*}$ as a function of available parental care (*r*_0_), initial juvenile size (*L*_0_) and juvenile mortality (*μ*_J_), with no consideration for costs to the parent. If adult mortality in the absence of offspring was large in comparison to additional adult mortality related to offspring we expect this would be close to the optimal strategy for the population, since there would be very little advantage to be gained by a parent holding out for future reproductive success over the present. As expected, this result fits the general theory and observations of brood size [1,26]:

— As juvenile mortality increases the optimal brood size decreases.
— As juvenile growth rates decrease, e.g. through lower food availability, the optimal brood size decreases.

This result assumes that the parental advantage conferred to offspring is an increased juvenile growth rate. If we assume parents can confer lower juvenile mortality to smaller broods, e.g. as a result of parental care, then *μ*_J_ = *μ*_0_*B*_0_ and we see the same result.

## 4. Adult survival and mortality

We combine the key output of the juvenile model with another relatively simple model of adult mortality. We assume that having offspring is detrimental to the adult through higher mortality (trading survival/maintenance investment for reproductive output) and increasing the number of offspring increases the adult mortality rate. As the number of offspring increases, the probability of an adult surviving to the next breeding season decreases to zero. For clarity we assume that breeding seasons are annual but the model easily generalizes to longer or shorter times between breeding seasons provided juveniles reach maturity before the next breeding season begins. These assumptions make adult mortality a nonlinear and increasing function of offspring. We model this using the adult mortality function

4.1 $$\mu_{A}=\;1-(1-\mu_{A0})\exp(-\alpha B_{0}),$$

where *μ*_A0_ is the intrinsic adult mortality rate, i.e. the probability of an adult dying each year if they have no offspring. The effect of brood size on adult mortality is *α*, referred to as brood cost mortality. If there is no increase in adult mortality associated with increasing brood size, i.e. *α* = 0, then, using the example parameters of table 1, with *μ*_A0_ = 0.07 an individual has an expected lifespan of 14 years regardless of offspring. If brood cost mortality is low, e.g. *α* = 0.1, adult mortality increases slowly as the number of offspring increases, e.g. with one offspring *μ*_A_ ≈ 0.16 and by 10 offspring *μ*_A_ ≈ 0.66 and the adult is unlikely to survive for two years. Conversely, adult mortality may increase quickly with the number of offspring, e.g. if brood cost mortality *α* = 1 having just a single offspring increases adult mortality to *μ*_A_ ≈ 0.66 and having five or more offspring carries an adult mortality rate greater than 0.99.

This particular choice of adult mortality function means that when brood size is small an additional offspring will have a larger detrimental effect on adult mortality but at larger brood sizes an additional offspring will have a much smaller effect; for example, increasing from one offspring to two in a single brood is a relatively expensive choice in terms of adult mortality but increasing from four to five offspring is far less costly. Other choices of adult mortality could include a more threshold type effect (e.g. a logistic function) where the effect on adult mortality of increasing offspring numbers was minimal for low numbers of offspring but started to increase rapidly around some threshold value. Although interesting and relevant this adds an additional parameter to our model and another layer of complexity not explored here.

## 5. Deterministic population model

For many species, reproduction happens at discrete intervals, sometimes, though not necessarily, annually, with a development time preceding that (e.g. for gestation or seed development). This results in a discrete time map for the population where each year or breeding season, adults have different mortality rates depending on the number of offspring that were born during that interval.

We now consider a very simple population model with discrete breeding seasons where adults may survive and reproduce for many years. Each year the population is composed of surviving individuals from the previous year and their offspring that reached maturity. As before we assume that the time between breeding seasons is a single year for annual reproduction but could also be shorter for species that breed more frequently. The adult mortality rate *μ_A_* depends on the brood size, *B*_0_, and, on average, the probability an individual will survive to the next year is 1 − *μ*_A_(*B*_0_), i.e. 1 – the adult mortality rate. The expected number of surviving offspring per adult, *B*_∞_ also depends on the brood size. There is no limiting effect of population size due to competition, i.e. the population is relatively small in relation to the environmental capacity. This gives the discrete time map for the number of adults alive in year *G* as

5.1 $$N_{G+1}=(1-\mu_{A}(B_{0}))N_{G}+B_{\infty }(B_{0})N_{G}.\;$$

This is a simple linear map with a population growth rate of *R*_0_ = 1 − *μ_A_*(*B*_0_) + *B*_∞_(*B*_0_).

Using the adult mortality model (8) and offspring model (6) derived above our population model is

5.2 $$N_{G+1}=(1-\mu_{A0})\exp(-\alpha B_{0})N_{G}+B_{0}\exp\left(-\frac{\mu_{J}B_{0}(1-L_{0})}{r_{0}}\right)N_{G}.$$

The population growth rate *R*_0_ is

5.3 $$R_{0}=(1-\mu_{A0})\exp(-\alpha B_{0})+B_{0}\exp\left(-\frac{\mu_{J}B_{0}(1-L_{0})}{r_{0}}\right).\;$$

**Figure 1.**
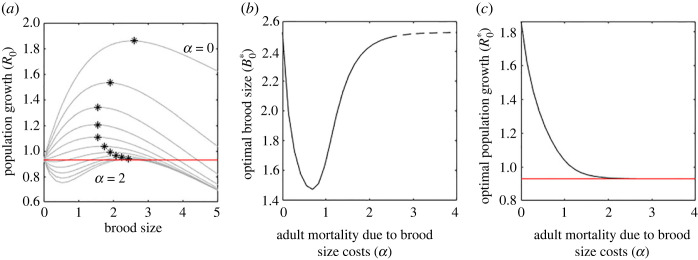
Optimal brood size changes as ***α***, the effect of brood size on adult mortality, varies. (*a*) Population growth rates for a range of brood sizes and brood cost mortality, *α*, stars mark the optimal brood size at each *α*. (*b*) Optimal brood size falls as brood cost mortality (*α*) increases from zero towards 1, then rises again. For extremely high values of brood cost mortality (*α* ≫ 2, dashed line), the optimal brood size is the same as for individuals with no brood cost mortality. (*c*) The optimal population growth rate is negative ($R_{0}^{*}<1$) for high (*α* > 1) brood cost mortality. The red line (panels *a* and *c*) shows the population growth rate with no reproduction, i.e. *B*_0_ = 0. All parameters as in table 1.

Figure 1*a* shows the population growth rate at different brood sizes for a range of brood cost mortality, *α*, from 0 to 2. Now the optimal brood size, $B_{0}^{*}$, (marked as * for each value of brood cost mortality, *α*, shown) is the brood size which optimizes population growth rather than just offspring numbers. When having more offspring does not increase the overall adult mortality *μ*_A_, (top line, *α* = 0) the optimal population strategy is the offspring optimal strategy (table 1 for example parameter values used). Increasing *α* results in a lower brood size being optimal, up to a point (figure 1*b*). This represents the trade-off of improving the probability of the parent surviving and reproducing in future years and having more surviving offspring this year. As *α*, the brood cost mortality, increases (figure 1*b*, just under *α* = 1) the strategy switches and the optimal brood size increases as it becomes more advantageous to have more offspring despite the high costs. This is because adult mortality is almost guaranteed regardless of the number of offspring. As *α* increases further and becomes very high (around *α* = 2.5) even this strategy fails because large numbers of offspring also result in higher juvenile mortality and the best strategy for the population is to have no offspring in order to maximize the likelihood of the parent surviving. Mathematically, in this region the population growth rate has a local maximum for a non-zero brood size (figure 1*b* dashed line) but the corresponding population growth rate matches that for no offspring. Such a strategy would never evolve if implemented as a deterministic process, since if the same strategy is used in every breeding season, the population must shrink rather than grow. The population growth rate at the optimal brood size is shown in figure 1*c*. As the brood cost mortality, *α*, increases the population growth rate falls. For even moderate effects on adult mortality (*α* ≈ 1) the population growth rate is below 1 regardless of brood size, indicating a threshold for the maximum brood cost mortality likely to arise in a real-world scenario. Alternative parameter sets are shown in the case studies.

## 6. Stochastic models

We consider the effect of stochasticity on optimal brood size in two ways: individual and environmental variation. Individual variation allows brood size to vary between individuals in the same year or breeding season according to some distribution, e.g. Poisson, which may affect the optimal mean brood size. Environmental variation assumes that the environmental conditions can vary across years but all individuals in that year will have the same number of offspring. We use the example of food availability being driven by mast seeding events.

### 6.1. Individual variation

If we allow the brood size across individuals within a single year to vary according to some probability distribution, the population now follows a stochastic process. In the deterministic model we defined adult mortality at the population level, i.e. the proportion of the population that would die each year. In a model with individual variation this becomes the probability that an individual will die each year. The adult mortality is conditional on the number of offspring they produce

6.1 $$P(\;\mathrm{Adult}\;\mathrm{dies}\;|\;B_{0})=\;1-(1-\mu_{A0})\exp(-\alpha B_{0}),$$

and the juvenile mortality is conditional on the brood size they were born into

6.2 $$P(\;\mathrm{Offspring}\;\mathrm{dies}\;|\;B_{0})=\;1-\exp\left(-\frac{\mu_{J}B_{0}(1-L_{0})}{r_{0}}\right).$$

The number of offspring produced by an adult is a Poisson distributed random variable with mean *B*_0_.

**Figure 2.**
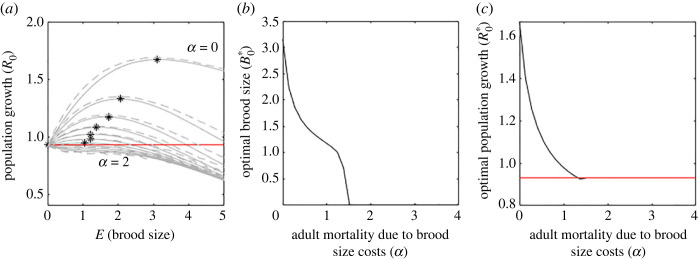
With individual variation the optimal brood size drops to zero at lower brood cost mortality, ***α***, than in the deterministic case. (*a*) Population growth rates for a range of brood sizes and brood cost mortality, *α*. Dashed lines show outputs of the stochastic process, solid lines are from the approximating stochastic map. Stars show the highest population growth rate at the optimal brood size as given by the stochastic map. (*b*) Optimal brood size for a range of brood cost mortality, *α*, using the approximate stochastic map. For high *α*, having no offspring is the most advantageous strategy for the population. (*c*) Corresponding optimal population growth rates for increasing brood cost mortality, *α*. Red line (*a* and *c*) shows the population growth rate with no reproduction.

Although this stochastic process has no analytical solution it can be approximated as a stochastic linear map identical to the deterministic map used earlier but now the parameter *B*_0_ follows a Poisson distribution with mean *B*_0_. This stochastic linear map is only an approximation to the population growth as it assumes that all individuals in the population that year have a defined number of offspring and that number varies across years, rather than allowing individuals within a single breeding season to vary their offspring number. However, it does have an excellent match to the stochastic process (figure 2*a* comparison of dashed and solid lines). In the one-dimensional stochastic linear map, the population growth rate, *R*_0_, is no longer the eigenvalue but rather the Lyapunov exponent which gives a time average of the population growth at each iteration of the map. Provided the random variable is IID, the population growth rate is

6.3 $$R_{0}=\exp\left(\;\overset{\infty }{\underset{\;j=0}{\sum }}\log\left((1-\mu_{A0})\exp(-\alpha j)+j\exp\left(-\frac{\mu_{J}j(1-L_{0})}{r_{0}}\;\right)\right)f(j)\right),$$

where *f*(*j*) is the probability of having *j* offspring. Figure 2*a* shows the results of the stochastic process (averaged over 100 simulations) and the approximating map. With individual variation (using the approximating map) we see very similar results to the deterministic case but with overall lower population growth (figure 2*c*). The key difference is that there are no intermediate values of *α* where the optimal brood size increases. The switch to no offspring occurs at a much lower brood cost mortality (figure 2*b*) and the best population growth rate is slightly lower (figure 2*c*).

### 6.2. Environmental variation

We now consider the case where the environment changes each year. Starting with the deterministic linear population growth map derived earlier, we assume that the available resources fluctuate between years. This could be from simple climatic variation either changing the available resources or requiring more energy to be spent on self-maintenance or could be more extensive variation due to e.g. mast seeding. The juvenile growth rate *r*_0_ is now a random variable *r_Y_* with distribution, $r_{Y}{\tilde{f}}_{r}(x)$. Again the Lyapunov exponent of the map gives the population growth rate

6.4 $$R_{0}=\exp\int \log\left((1-\mu_{A0})\exp(-\alpha B_{0})+B_{0}\exp\left(-\frac{\mu_{J}B_{0}(1-L_{0})}{X}\right)\right)f_{r}(X)dX.$$

We let *r_Y_* follow a lognormal distribution with mean *r* and variance *σ*^2^. This distribution is chosen to represent food availability in a masting climate which has been seen to follow a lognormal distribution. Similar results can be seen using other positive distributions of juvenile growth rates, e.g. Gamma. As the mean and variance are independent we hold the mean constant so the model output is comparable to the deterministic case with *r*_0_ = *E*(*r_γ_*) (i.e. no long-term trend) and vary the coefficient of variation, CV=σ/r, by altering the variance to model different strengths of environmental variation. Using these definitions, the strength of the environmental stochasticity can now be thought of as the coefficient of variation (CV) of a mast seeding environment. Note that the brood produced each year by an individual parent remains the same size, i.e. we do not allow for plastic adjustments in brood size according to resource availability. However, the optimal brood size in a variable environment will take into account the full range of long-term environmental variation. This optimum may therefore differ from that found in the model with individual variation.

Another candidate parameter for change under environmental variation is the adult mortality. At this stage we have assumed this remains the same even in high food years. The case study presented later gives an example where adult mortality is affected by environmental variation separately of the effect of increased offspring.

**Figure 3.**
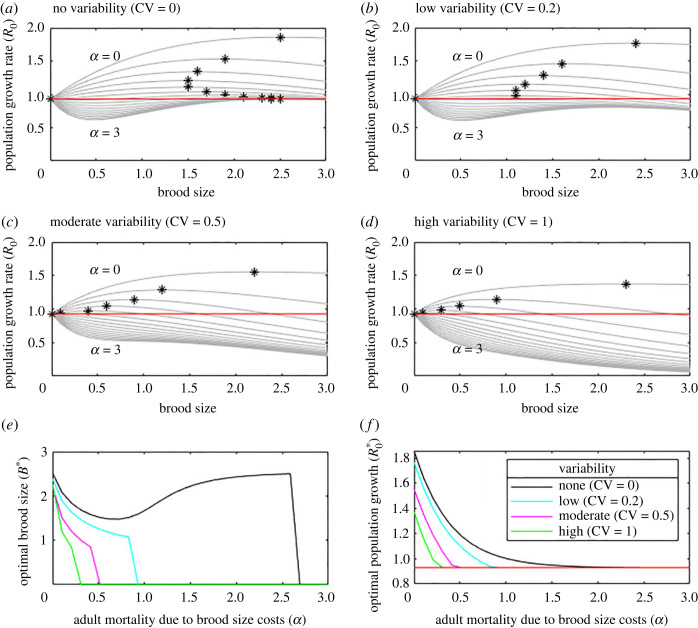
With a constant brood size across years, increasing environmental variation decreases the population growth rate. Brood size against population growth rate for a range of brood cost mortality *α* (highest line *α* = 0, lowest *α* = 3) for four different levels of environmental variability (*a–d*). The optimal brood size is marked with a star for each case. (*e,f*) summarize the optimal brood size and population growth rate. In all cases, very high adult mortality due to brood size costs, *α*, means the optimal brood size is zero (*e*). Red line (*a,b,c,d* and *f*) shows the population growth rate with no reproduction.

Figure 3 shows how the population growth rate depends on brood size mortality from *α* = 0 to *α* = 3 for four different fixed levels of environmental variation. When there is no environmental variation (figure 3*a*) the optimal brood size initially decreases with brood cost mortality, *α*, then increases before dropping to zero. These results are identical to those in figure 1. When environmental variation is low (figure 3*b*) we see a similar picture but there is no subsequent increase in optimal brood size as *α* increases. This pattern continues for higher variation (figure 3*c* and *d*) where the drop occurs before the optimal brood size increases. Figure 3*e* and *f* summarize these results showing the optimal brood size and corresponding best population growth rate as *α* is varied for different levels of environmental variation. If brood size is constant from year to year despite annual variations in the environment the optimal brood size (figure 3*e*) and the associated population growth rate (figure 3*f*) decrease as environmental variation increases.

**Figure 4.**
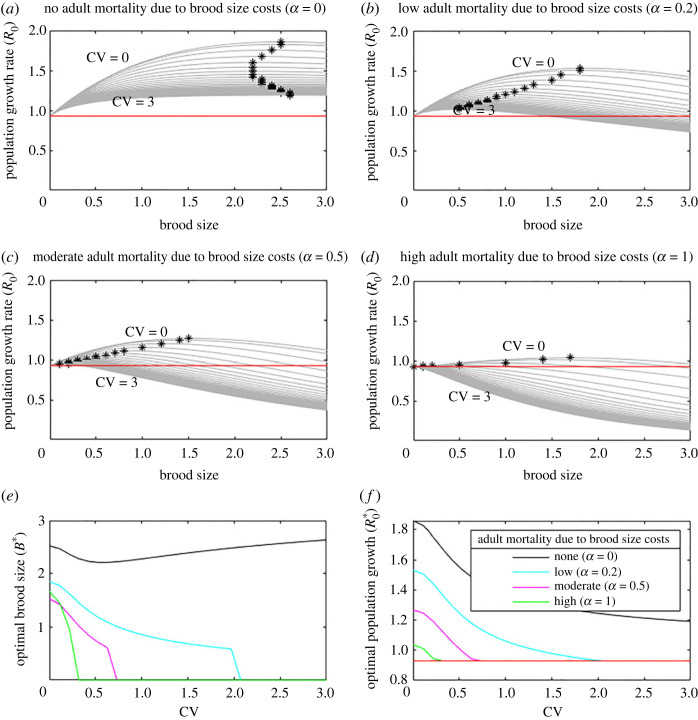
In a variable environment, the optimal brood size strategy varies but overall population growth is decreased. The optimal brood size (stars in *a–d*, and as a function of resource coefficient of variation, CV, in panel *e*) and resulting population growth rate (y-axis, panels *a–d*, and as a function of CV in panel *f*) are shown against CV for a range of *α*. If having offspring results in any increased adult mortality, i.e. *α* > 0, the population growth rate decreases with increasing CV (*b–d, f*), and if the brood cost mortality is high enough, there is a threshold CV above which zero reproduction is optimal (*e*). Red line (*a,b,c,d* and *f*) shows the population growth rate with no reproduction.

In figure 3 we presented results across many range of values of brood cost mortality for a limited range of environmental variation levels, i.e. CV. In figure 4 we consider the same results but present them for many values of environmental variation at limited values of brood cost mortality. This alternative viewpoint of the same results sheds further light. If *α* = 0 and there is no effect of offspring on adult mortality (figure 4*a*), increasing variation in resource availability reduces the population growth rate but only has a small effect on the optimal brood size, with a minimum at intermediate CV. However, when *α* > 0, increasing the CV reduces both the population growth rate and the optimal brood size (figure 4*b–d*). As *α* and CV increase, the optimal brood size switches to zero (figure 4*e*) and the population growth rate tends to an overall decrease corresponding to the intrinsic adult mortality rate without reproduction, *μ_A_*_0_ (figure 4*f*). As a result, we should not expect to find species with such high reproductive costs in such variable environments, as they would not be viable. However, species adapted to variable environments but whose costs have risen, e.g. due to land-use change, or who have high costs but are now experiencing higher variability in resource availability, e.g. due to climate change, may now face a catastrophic population collapse due to an inability to support reproduction during years with poor resource availability.

### 6.3. Synchronizing reproduction and environmental variation CV in masting

A prominent feature of some species living in areas with large environmental variations is that breeding cycles synchronize with the environment using a plastic strategy. Many plant species have seeding habits that are linked to variable climate cues such as temperature (e.g. Kelly *et al*. [16]) and precipitation [27]. This then filters through trophic levels as variable seed production supports consumers. For example, kākāpō (*Strigops habroptilus;* a large, flightless parrot) sporadically reproduce in years in which rimu (*Dacrydium cupressinum*; a mast seeding conifer in New Zealand) has a large seed crop [19]. This strategy allows individuals to take advantage of years with high resource availability to raise a bigger brood and to prioritize adult maintenance and survival in years when resources are scarce.

We now adapt the model to assume that resources fluctuate annually affecting the growth rate of juveniles (as previously) but now the parent has the optimal number of offspring each year depending on the food availability rather than having the same number each year. Once again the Lyapunov exponent of the map gives the population growth rate

6.5 $$R_{0}=\exp\int \log\left((1-\mu_{A0})\exp(-\alpha B_{0}^{*})+B_{0}^{*}\exp\left(-\frac{\mu_{J}B_{0}^{*}(1-L_{0})}{X}\right)\right)f_{r}(X)dX.$$

Using mast seeding as an example food availability follows a lognormal distribution [16]. We assume, as previously, the juvenile growth rate is also lognormally distributed.

**Figure 5.**
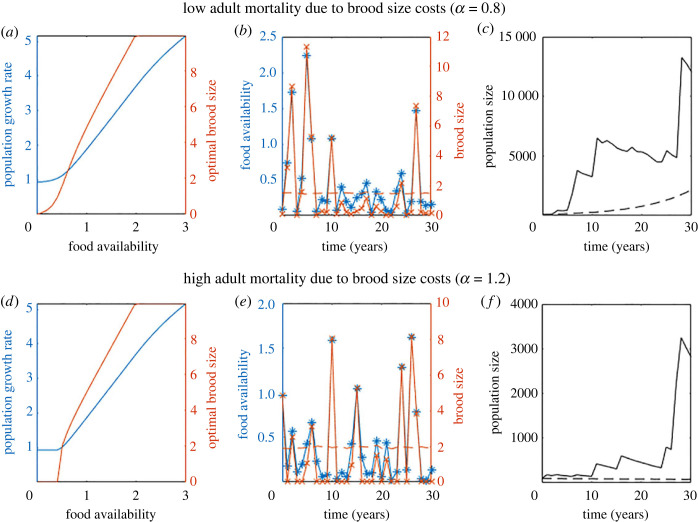
When resources vary population growth is higher when individuals can synchronize reproduction with resource availability. (*a–c*) Results for a lower brood cost mortality, (*d–f*) results for a higher brood cost mortality. (*a* and *d*) Population growth (blue) and optimal brood size (orange) for a range of food availabilities. (*b* and *e*) Example time series of variable resource availability (blue) and synchronized optimal brood size with a plastic strategy (orange). The orange dashed line shows optimal brood size under average food availability. (*c* and *f*) Corresponding time series of the population size over time when brood size synchronizes with food availability (solid line) and when brood size is constant across years (dotted line). Food availability is lognormally distributed, *CV* = 1, all other parameters as in table 1.

Two examples of the population growth under stochastic resource availability and corresponding plastic optimal brood size trajectory are shown in figure 5. In high seed years individuals have many offspring but in low seed years the optimal number of offspring is zero to minimize adult mortality (figure 5*a* and *c*). For both values of brood cost mortality the optimal number of offspring if brood size is constant, based on the expected food availability, is close to 2 (figure 5*b* and *d* dashed orange line). Figures 5*c* and *f* show time series of the population size for the optimal constant brood size (dashed line) and the brood size using a plastic strategy synchronized with food availability (solid line). For lower brood cost mortality the population growth is positive under both the plastic strategy and the constant strategy (figure 5*c*), though for the period shown here the plastic strategy is faring better. When brood cost mortality is higher (figure 5*f*) the plastic strategy is allowing the population to grow whereas the constant strategy shows population decline.

**Figure 6.**
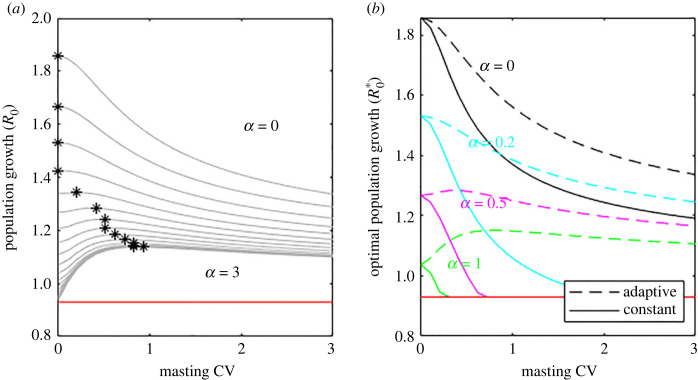
The ability to synchronize reproduction with environmental variation can lead to improved population growth in a variable environment. (*a*) Population growth using a plastic strategy synchronized to food availability for a range of brood cost mortality, *α*, and environmental variation, CV. Stars mark the optimal level of environmental variation. (*b*) Comparing a plastic strategy that synchronizes with the varying environment with a constant one. For all values of brood cost mortality a plastic strategy (dashed lines) outperforms a constant strategy (solid lines). When having more offspring has a strong effect on adult mortality (pink and green lines) the ability to synchronize breeding cycles with mast years (CV greater than 0) can improve population growth relative to the best strategy in a constant environment (CV = 0). Red line shows the population growth rate with no reproduction.

When brood cost mortality is high it is advantageous to live in a highly varying environment. The population growth using an optimal plastic strategy as the coefficient of variation of food availability increases is shown in figure 6*a*. When there is no brood cost mortality (top line*α* = 0) environmental variation, controlled by the masting CV of the food supply, is detrimental to population growth. In this case the optimal environment (marked *) is a constant one. As brood cost mortality increases further, (at *α* ≈ 0.5) the deterministic environment is no longer the best for population growth and the * moves away from the *CV* = 0 vertical axis. At this point a small amount of stochasticity and the ability to react to this by varying brood size is advantageous. This unintuitive result, which occurs even though the mean available resources in the varying environment match the annual resources of the deterministic environment, comes about because the boom in reproduction that is the consequence of a good year outweighs the downside of a bad year. In the deterministic environment all years result in fairly low reproduction. Adapting to annual variation means that the bad years are only slightly worse but the good years are excellent, outweighing this small decrease. Mathematically this is a consequence of Jensen's inequality applied to the mean of a convex function of a random variable. If brood cost mortality is very high a plastic strategy and a highly varying world are the best combination. A brief sensitivity analysis shows that if adult mortality is lower, i.e. adults are very long lived (such as for many tree species), the optimal level of environmental variation increases.

Finally, we compare a plastic strategy with a constant strategy directly (figure 6*b*). The dashed lines are a repeat of the equivalent line in figure 6*a* representing the optimal growth rate for a population with a plastic strategy. The solid line gives the population growth rate for individuals unable to adapt using environmental cues and are following a constant strategy. For any level of environmental variation and any brood cost mortality a plastic strategy is more advantageous than a constant one. As expected the advantage is largest at higher environmental variation. What is less intuitive is that when the parental cost of having offspring is high a highly varying environment and the ability to adapt to it can lead to higher population growth than the best strategy in a constant environment.

## 7. Case study

We construct a thought experiment in which we consider and contrast two species. Both species have the same intrinsic adult mortality rate, *μ_A_*_0_ = 0.1 (10-year lifespan), juvenile mortality rate, *μ* = 0.1, and the same offspring size, *L*_0_ = 0.01. For ease of understanding we refer to our two species as a kākāpō and a blackbird as they share some characteristics of these two species but the example is not meant to be a literal representation of these species.

Our first species is represented by the kākāpō. It lives in a highly varying world with good (high resource availability) and bad (low resource availability) years, cf. the mast-driven beech forests of Fiordland, New Zealand. The probability of a year being good is *p*_mast_. In a good year juvenile growth rates are high *r*_good_, in a bad year juvenile growth rates are low *r*_bad_. In a good year there is no brood cost mortality, so *α*_good_ = 0. In a bad year there is a high brood cost mortality, *α*_bad_ = 0.5. If *r*_good_ = 0.2 and *r*_bad_ = 0.05 the respective optimal brood sizes are 2.02 and 0.19 with population growth rates 1.64 and 0.95. If *p*_mast_ = 0.25 the expected overall population growth rate is 1.09.

Our second species is represented by the blackbird. It lives in a constant world, cf. a temperate, agricultural landscape subsidized by suburban garden feeding. The juvenile growth rate is the average of *r*_good_ and *r*_bad_, weighted by the relative frequency of each year type *p*_mast_. We choose *α* such that the overall population growth rate is the same as for the blackbird. For example, if *r*_good_ = 0.2, *r*_bad_ = 0.05 and *p*_mast_ = 0.25 we choose to be *α* = 0.29 and the resulting optimal brood size is 0.78 to give the same expected overall population growth rate of 1.09.

We then consider the consequences of the two species moving between environments, either as a result of climate change or relocation. We assume the masting-evolved kākāpō responds to environmental cues that trigger masting events in its native environment but now the environment doesn't follow suit. Individuals will continue to switch between brood sizes with the same probability but the juvenile growth rate and adult brood cost mortality will now be those associated with the constant environment of the blackbird. This move to a constant environment results in a lower overall population growth rate of 0.95, a reduction of almost 13% on the average population growth rate, but no worse than the population growth rate in bad years of its original environment.

Similarly, we consider the consequences of the garden-fed blackbird, moving to the boom and bust environment of the kākāpō. We assume our blackbird does not notice the environmental cues and continues to have its evolved optimal brood size each year. When this is combined with the highly varying juvenile growth rates and adult mortality experienced by the kākāpō this results in an overall population growth rate of 0.96 or a reduction of 12% on the population growth rate under constant conditions.

Under this particular set of parameter values both species experienced a reduced overall population growth rate when they moved to the other environment and the blackbird was slightly worse off than the kākāpō. Is this true for all parameter values? Is the environmental change always detrimental and is the detrimental effect similar for both species?

**Figure 7.**
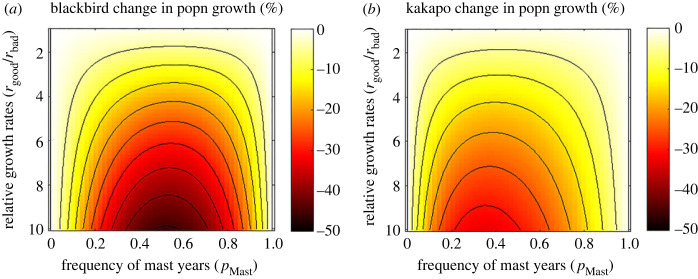
The effect of different environments on population growth. (*a*) The relative effect on the population growth rate of a blackbird like species from an environment with little annual variation moving from a constant to a varying environment. (*b*) The relative effect of a masting evolved species like the kākāpō moving from a variable to a constant environment. Other parameters are *μ*_A0_ = 0.1 and *r*_bad_ = 0.05. Black lines show contours.

Figure 7 shows the effect of moving between environments for a range of parameter values. At each parameter set we hold *r*_bad_ = 0.05 and change r*_good_* (this is shown as the relative value *r*_good_/*r*_bad_ on the vertical axis). We consider a range of masting frequencies from 0 (always a bad year) to 1 (always a good year). In the masting environment the brood cost mortality in a bad year is always *α*_bad_ = 0.5, in a good year there is no brood cost mortality, *α*_good_ = 0. As in the example we calculate the expected population growth rate of the kākāpō in the masting environment and chose *α* in the constant environment to give a matching population growth rate for the blackbird in the constant environment. At every parameter set the overall population growth rate is different but population growth of the kākāpō in the masting environment matches the population of the blackbird in the constant environment.

Figure 7*a* shows the expected percentage change in population growth rate for the blackbird when it is moved to the masting environment. We immediately see that the move never has a positive effect. When the ratio of *r*_good_/*r*_bad_ is close to 1, i.e. there is almost no difference between a good year and a bad year in the masting environment, the change in population growth rate caused by the move in environment is minimal. As the boom and bust conditions of masting become more extreme, i.e. *r*_good_/*r*_bad_ increases, the detrimental effect of this highly varying environment on the blackbird increases. The exception here is when the masting frequency is very high (*p*_mast_ ≈ 1) or very low (*p*_mast_ ≈ 0). In both of these cases the masting environment is almost always good or always bad and effectively constant. The decrease in population growth rate is relatively symmetrical in the mast year frequency and the detrimental effect is highest when *p*_mast_ ≈ 0.5.

Conversely, figure 7*b* shows the effect on population growth rate for the masting evolved kākāpō when it moves to the more constant world of the blackbird. Overall, we see the same effect: the bigger the difference in the juvenile growth rates between good and bad years, the larger the drop in average population growth rate when the species is moved to the constant environment. Now however, the biggest drop is not seen when there is a 50% chance of a good year but when good years are much less frequent than bad years. We also see that, overall, the move is not as detrimental to the kākāpō as it was to the blackbird when considering the equivalent new environment.

## 8. Sensitivity analysis

The results in the model apply to a wide range of parameter values. However, there is likely to be some sensitivity to the exact format of the model chosen. Our model choices were made for pragmatic reasons of simplicity and to give broad agreement with observations. We would expect the qualitative results to be robust to model details provided the same observations were considered. For example, we would expect little change from alternative models of juvenile growth and mortality, provided that an optimal brood size exists and is similarly affected by changes in juvenile growth rates and mortality.

A more sensitive choice is the model of the effect on adult mortality of having more offspring. Here we have chosen a function that quickly penalizes having offspring, particularly for increases at the lower brood sizes, i.e. the biggest change in adult mortality is always when increasing from 0 to 1 offspring. An obvious extension would be to change this function to something with a threshold, e.g. a logistic function, where there was some value of brood size where costs increased dramatically for the adult. We speculate that there would still be areas of parameter space that would result in a stochastic environment being beneficial to population growth (figure 6).

Our thought experiment focuses on changing the juvenile growth rates, via *r*_good_, and masting frequency, via *p*_mast_. It also shows an example where the environmental stochasticity has an effect on adult mortality rate by significantly reducing the effect of offspring during a good year. Sensitivity analysis of the other parameters showed that choosing a different value of *r*_bad_ (juvenile growth rate in bad years) had very little effect, providing the ratio *r*_good_/*r*_bad_ was considered. Changing the brood cost mortality in the masting environment *α*_good_ and *α*_bad_ also had very little effect, though a smaller difference between the two effects resulted in a smaller drop in population growth rate.

## 9. Discussion

Our simple model of juvenile growth and mortality shows that if a larger brood size results in lower parental care and a reduced juvenile growth rate or higher juvenile mortality then there is an optimal brood size to maximize the number of offspring that reach maturity. We coupled this juvenile growth model with a population level model that includes parental costs of increased adult mortality for larger brood sizes. As the effect of brood size on adult mortality increases, the optimal brood size varies. Individual variation, where brood size of individuals in the population in each year is distributed around the optimal brood size, results in lower population growth compared to the deterministic model.

If the environment changes dramatically between breeding seasons, but a species continues to have the same brood size each year regardless, then the optimal brood size decreases. Species then produce broods that are far below the optimum size in resource rich years and closer to the size expected in low-resource years. One real-life example of this is seen in red squirrels (*Sciurus vulgaris*), whose litter sizes were similar to the optimum during non-mast years, but were well below optimum litter sizes during resource-rich mast years [28], meaning that there is a quantifiable missed opportunity for population growth in these years.

Individuals can offset the detrimental effect of variable environments on overall population growth with a brood size strategy that varies depending on the resources available. Changing the size of brood to match the available resources is always more favourable to overall population growth than the best constant brood size strategy. This is seen in multiple species native to variable environments, from diverse plants [16,29] to rodents [30], birds [19] and giraffes [31].

Having more offspring is associated with higher parental costs through higher parental mortality or investment, in plants [11,32] and animals [12,33]. When other parameters are held equal, then higher population growth can be achieved in a variable world using a strategy that synchronizes brood size with food availability, compared to living in a constant world. This is because the costs of having offspring are avoided in all years when resources are not available to offset these. These strategies may benefit, or result in, particularly long-lived species (such as trees) who have the facility to ‘wait it out’ until the next resource pulse. However, if reproduction has a long lead-in time (such as for plants with multiple years between bud initiation and seed maturity [29]), the species must be sensitive to environmental cues to avoid false alarms. Our model does not currently incorporate non-perfect environmental perception, and this is an area for future development. Similarly, gender differences in parental costs may affect population growth rates; female organisms bear the majority of production costs in plants and animals [12,32] but male animals may experience higher reproductive competition costs [33].

We used a theoretical example of two species with the same overall population growth rate, one with a constant brood strategy for a constant environment, and one with a plastic strategy for a variable environment. We explored what happens if either of these species was moved into the alternative environment. In both cases the move is detrimental but often the losses are smaller for the species with the now defunct plastic strategy. This suggests that species who are adapted to synchronize reproduction with resource availability are likely to cope better with a changing environment as a result of climate change or human impacts, compared to those who have adapted to a specific set of conditions. In our case study, blackbirds were relatively unaffected when few years were resource poor (e.g. *p*_mast_ = 0.9), but as the frequency of bad years increased, overall population growth was significantly reduced, and to a greater extent than our kākāpō comparison. As environments become more variable, species who have already adapted to plastically adjusting their brood sizes in response to environmental conditions still have the problem of effectively detecting interannual cues, but they may be in a better position than those who only respond to seasonal cues [34] or none at all. We may then see a shift towards communities more dominated by species with variable strategies with a consequential effect on network interactions, *cf.* the movement towards generalist versus specialist species [35]. These findings align well with intuition in one way, e.g. theoretical expectations are that increasing variability is more difficult to cope with than decreasing variability, see for example [36]. However, typical intuition also predicts that specialists should have a much harder time coping with change than generalists [37]. At face value our results do not support this; our generalist blackbird fared worse under change than the specialist kākāpo. However, if we hypothesize that actually the kākāpo's strategy is more general because it can cope with a wide range of fluctuating environments, one of which is the minimally fluctuating environment of the blackbird, it once again aligns.

Invasive species that can respond to a changing environment might also have a competitive advantage over native species who cannot. For example, red squirrels in the UK appear to have a constant brood strategy [3]. Grey squirrels (*S. carolinensis*) in the UK have replaced red squirrels across much of the country, largely as a result of habitat- and disease-mediated competition [38]. However, it is not clear whether grey squirrels are better able to predict and respond to high resource availability relative to red squirrels, or whether they simply appropriate those resources first (e.g. [39]). Some squirrels do have a variable environment-driven reproductive strategy, such as the Arizona grey squirrel, *S. arizonensis* [40]. The native range of the UK's invasive grey squirrels is deciduous woodland in eastern North America, dominated by mast-seeding species such as oak and hickory [41], while red squirrel occur predominantly in conifer woodland across its Eurasian range [42]. The fact that red squirrels have survived longest in large, conifer-dominated woodland in the UK may not only be an indicator of that habitat's lower attractiveness to grey squirrels, but also the relative competitiveness of the two species in environments with differently varying resources; indeed, forest management in the UK designed to create ‘natural strongholds’ for red squirrels focuses on diverse coniferous tree species with different masting regimes specifically to reduce the likelihood of wholesale seed failure and even out the food supply [43].

Finally, we have explored how the optimal brood sizes differ for species with different ‘fixed’ brood sizes and with individual variation around the population mean, in constant and variable environmental conditions. We used simple parameters to represent costs in terms of mortality for adults and juveniles and reproduction, in order to derive our results. These could be developed further to explicitly represent mechanisms or attributes of functional groups, to explore in more detail how a changing environment might affect different aspects of specific species' population growth and/or community interactions. Some examples include hoarding of seeds by seed predators [44] or digestive efficiency [42]; individual seed size and viability versus brood biomass and how these relate to parental investment and offspring success; and cues driving bud initiation and maturation in plants [16,29] or reproduction in mammals [34].

Sæther & Engen [45] examined the general concept of evolutionary fitness in a fluctuating environment, in particular looking at how environmental variability affects the rate of adaptive change. Another next step for model development would be to add adaptive strategies within each model ‘run’. This would help shed light on the question of how quickly individual species may be able to adapt to changing environmental conditions in the real world, either after an abrupt change, e.g. due to introduction to a new location, or during a slower alteration such as warming temperatures or increasing variation in precipitation due to global change.

In conclusion, a reproductive strategy that can anticipate resources and vary optimum brood size to maximize population growth is of benefit to a wide variety of species in variable environments. However, the cost of predicting incorrectly is likely to be a crucial parameter that affects species ability to persist in a changing world, as a result of false negatives (lost reproductive opportunities) and false positives (reproduction without resources to support it), while interactions between species using different strategies may have large effects on community dynamics.

## Data Availability

This article has no additional data.
